# Hantavirus Cardiopulmonary Syndrome in Canada

**DOI:** 10.3201/eid2612.202808

**Published:** 2020-12

**Authors:** Bryce M. Warner, Sebastian Dowhanik, Jonathan Audet, Allen Grolla, Daryl Dick, James E. Strong, Darwyn Kobasa, L. Robbin Lindsay, Gary Kobinger, Heinz Feldmann, Harvey Artsob, Michael A. Drebot, David Safronetz

**Affiliations:** Public Health Agency of Canada, Winnipeg, Manitoba, Canada (B.M. Warner, S. Dowhanik, J. Audet, A. Grolla, D. Dick, J.E. Strong, D. Kobasa, L.R. Lindsay, G. Kobinger, H. Feldmann, H. Artsob, M.A. Drebot, D. Safronetz);; University of Manitoba, Winnipeg (B.M. Warner, J.E. Strong, D. Kobasa, L.R. Lindsay, G. Kobinger, H. Feldmann, M.A. Drebot, D. Safronetz)

**Keywords:** hantavirus cardiopulmonary syndrome, hantavirus pulmonary syndrome hantavirus, Sin Nombre virus, Canada, viruses, hantavirus

## Abstract

Hantavirus cardiopulmonary syndrome (HCPS) is a severe respiratory disease caused by Sin Nombre virus in North America (SNV). As of January 1, 2020, SNV has caused 143 laboratory-confirmed cases of HCPS in Canada. We review critical aspects of SNV virus epidemiology and the ecology, biology, and genetics of HCPS in Canada.

Sin Nombre virus (SNV; family *Hantaviridae*, genus *Orthohantavirus*, species *Sin Nombre orthohantavirus*) is the primary etiologic agent of a severe respiratory illness known as hantavirus pulmonary syndrome (HPS) or hantavirus cardiopulmonary syndrome (HCPS), of which »1,000 cases have been confirmed across North America. SNV was identified after an outbreak in 1993, and association with the deer mouse (*Peromyscus maniculatus*) was established (*1*,*2*). In North America, the case-fatality rate (CFR) of HCPS is 30%–35% (*3*). Transmission of SNV to humans occurs predominantly through direct contact with infected deer mice or their excreta in peridomestic settings (*4*). We summarize critical aspects of SNV biology and the epidemiology of infections in Canada over the past 25 years, including a phylogenetic analysis of SNV from different geographic areas over time. 

## The Study

Public health authorities in Canada follow the case definition for HPS/HCPS of the Pan American Health Organization (*5*). Samples from suspected hantavirus-infected patients are sent for diagnostic testing to the National Microbiology Laboratory, Winnipeg, Manitoba (Appendix). Several criteria must be fulfilled for laboratory confirmation of an HCPS case: the presence of hantavirus-specific IgM; a >4-fold increase in hantavirus-specific IgG from sequential samples; and a positive reverse transcription PCR (RT-PCR) amplification of hantaviral RNA or positive immunohistochemical staining for hantaviral antigen in the tissues. Detection of IgM or a positive RT-PCR amplification occurs most often in early clinical disease, whereas IgG is typically detected throughout infection, even in the prodrome. Detection of hantavirus-specific antibodies remains the primary diagnostic criterion (*6*,*7*). It is difficult to propagate virus from clinical samples; therefore, isolation is not routinely attempted.

Active surveillance of HCPS began in Canada in 1994; it became a nationally notifiable disease in 2000 (*6*). Surveillance of small mammals across Canada was initiated in 1994 and has since documented SNV-infected deer mice in all provinces except Prince Edward Island and Nova Scotia, and in Yukon Territory but not in the Northwest Territories or Nunavut (*6*). The prevalence of SNV among deer mouse populations varies spatially and temporally, typically 10%−30% (*6*,*8*).

**Figure 1:**
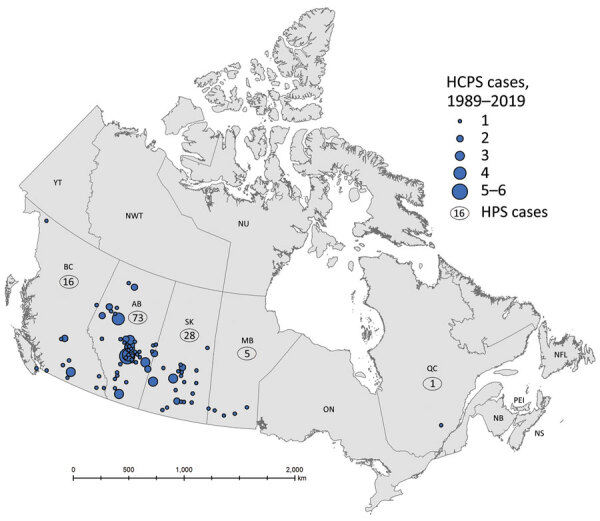
Geographic distribution of confirmed cases of hantavirus cardiopulmonary syndrome (HCPS) in Canada, 1989–2019. The map shows the locations of cases in Canada based on provinces where Sin Nombre virus infection was likely contracted; data were provided on diagnostic requisitions, physician reports, or follow-up investigations. Numbers in circles indicate number of cases for that province. The locations of 15 cases could not be mapped due to insufficient information.

**Figure 2:**
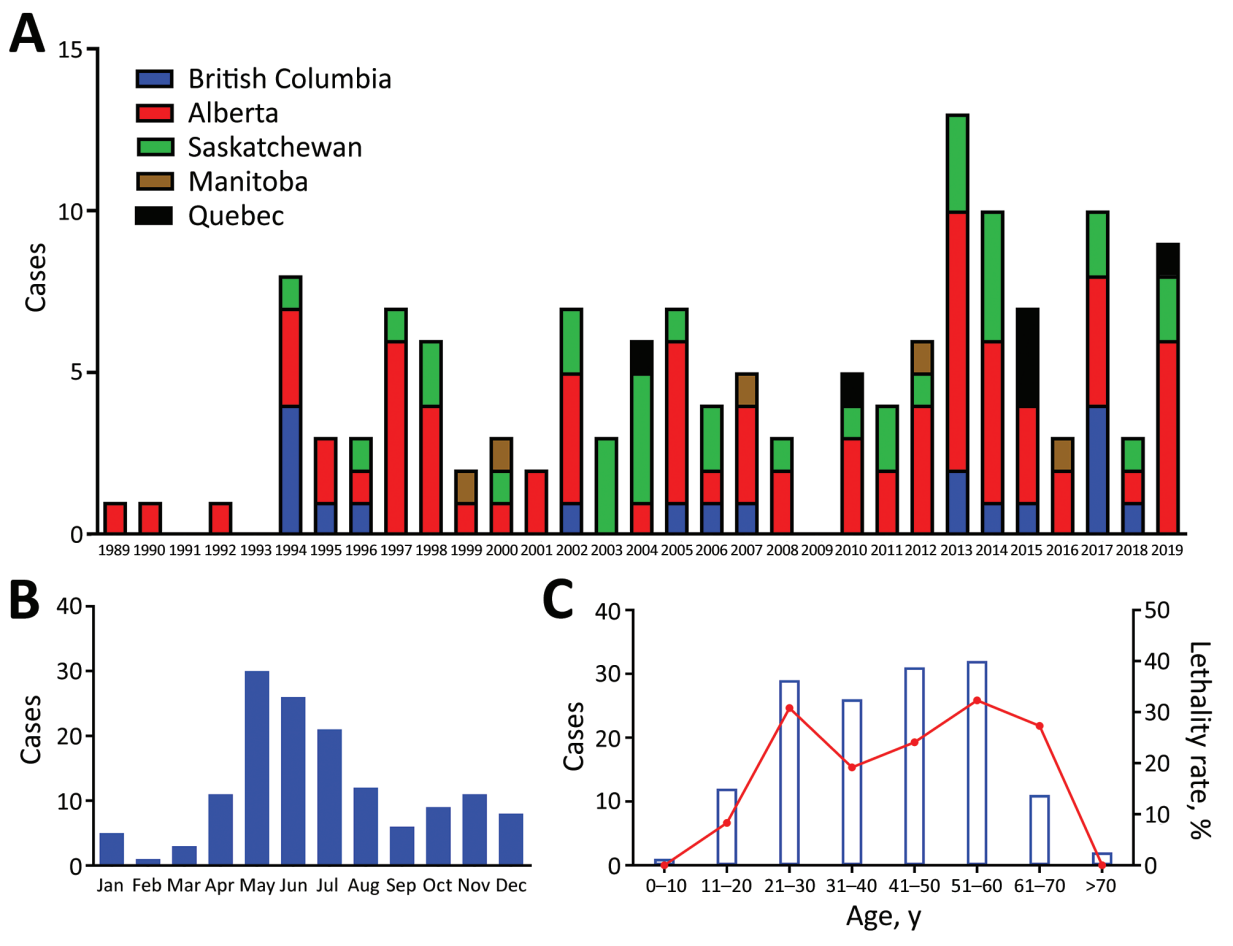
Cases of hantavirus cardiopulmonary syndrome (HPCS) by province, year, month, and patient age, Canada, 1989–2019. A) HCPS cases per year by province. B) Seasonal incidence of HCPS in Canada as shown by number of cases diagnosed in each month. A clear trend toward spring/summer contraction of disease can be seen. C) HCPS cases by age with associated case-fatality rate. Blue bars indicate the number of cases diagnosed in each age group; red line shows case-fatality rate associated with each age group.

As of January 1, 2020, a total of 143 cases of HCPS had been laboratory confirmed in Canada, including 3 cases retrospectively identified since 1993 that were diagnosed in 1989, 1990, and 1992 (Figure 1; Figure 2, panel A). Annually, an average of 4–5 cases (range 0–13 cases) are confirmed in Canada (Figure 2, panel A). Although cases of HCPS have been diagnosed in every month, they are most common in spring and summer (Figure 2, panel B), peaking in May−June and then gradually decreasing. Peaks in HCPS cases are likely driven by seasonal increases in deer mouse populations coupled with increased human contact with environments contaminated with SNV-infected rodent excreta. All cases except 1 have occurred in the 4 westernmost provinces of Manitoba, Saskatchewan, Alberta, and British Columbia; Alberta had the most cases (Figure 1; Figure 2, panel A). Most cases in these provinces have been in southern rural, often agricultural, settings. Most HCPS cases occur in geographic and spatial isolation, although clusters have been reported across communities and within households. The most northern case of HCPS in Canada was documented above the 59th parallel in British Columbia, <20 km from the border with Yukon Territory. Six cases have been diagnosed in patients from Quebec; 5 were directly attributed to military exercises or travel in western provinces (*9*,*10*). The sixth case-patient was a resident in the Nicolet-Yamaska municipality with no travel history except to a nearby recreational lakeside property. To date, this case remains the only HCPS case with autochthonous transmission east of Manitoba. The western bias observed in HCPS case distribution in Canada mirrors that reported in the United States (*7*).

The mean age of HCPS patients in Canada is 40; cases have occurred in all age brackets from 10–70 years of age (median 42, range 7–76 years of age) (Figure 2, panel C). Overall, the CFR of HCPS in Canada is 25% (34/137 cases; 6 outcomes unknown). HCPS occurs more frequently in male patients (99/143 cases). CFR is higher in female than in male patients, although the difference is not statistically significant (15/43 [35%] vs. 19/94 [20%]; relative risk = 1.726 [0.9733–3.060]; p = 0.0651 by χ^2^ test) (Table). Differences in infection outcome based on age and sex of patients is an area of research that could lead to insights into the pathogenesis of HCPS and, similar to immunological studies using patient samples, could provide a means of identifying important signatures associated with severe infection and poor outcome (*11*,*12*).

**Table:** Comparison of outcomes for confirmed cases of hantavirus cardiopulmonary syndrome in male and female patients, Canada

Sex	No. fatal cases	No. nonfatal cases	Relative risk (95% CI)	p value (χ^2^ test)
M	19	75	1.726 (0.9733−3.060)	0.0651
F	15	28		
Total	34	103		

When available, sequence data for HCPS cases in Canada appear to share a relatively recent common ancestor. This ancestor appears to have diverged from viruses from deer mice in eastern Canada (Ontario eastward) before diversifying into the western Canada (Manitoba westward) genotypes that have most commonly caused clinical disease in humans and are also found in deer mice from western Canada (Appendix Figure 1, panels A–C). The eastern Canada genotypes cluster with a clinical isolate from a case in New York, USA, suggesting that, despite a lack of cases in eastern Canada, these viruses may also be pathogenic to humans. 

For both the small (S) and medium (M) genomic segments, sequences from western Canada tend to group relatively tightly together and with S segment sequences from Montana and New Mexico. This evidence suggests that latitude does not account for the variation in the S sequences the way longitude does, with a clear separation of eastern and western genotypes. However, M segment sequences from western Canada are more closely related to samples from California, USA, suggesting some M segment variation in the north–south gradient (*13*). The biologic relevance of these variances remains to be investigated.

The median estimate of the average mutation rate for the S segment is 1.03 × 10^−3^ substitutions/site/year (95% highest posterity density interval 0.51–1.72 × 10^−3^ substitutions/site/year) whereas the estimate for the M segment is 3.79 × 10^−4^ substitutions/site/year (95% highest posterity density interval 1.82–6.76 × 10^−4^ substitutions/site/year). However, we were unable to distinguish whether this difference is an artifact of the modeling or the underlying data, due to limited datasets for S (150–200 nt) and M (300–400 nt) segments, or analyzing variables versus conserved regions. Because of the limited data inherent with small nucleotide fragments, we could not assess if differences are potentially due to reassortment or recombination or if differing selective pressures cause different mutation rates. 

On occasion non–SNV-related hantavirus infections have been diagnosed in Canada; however, studies on non-SNV hantaviruses within Canada are limited. Two imported HCPS cases from Argentina and Bolivia were detected in returning travelers by reverse transcription PCR analysis; amplicon sequencing determined the etiologic agent was Andes virus or a closely-related variant (*6*). In addition, a traveler returning from Siberia in 2002, who originally tested seronegative using an in-house ELISA, subsequently tested seropositive for Dobrava virus. A small proportion of coastal rats have shown seroreactivity for Seoul virus; however, human infections in Canada have been limited to a recent cluster associated with imported pet rats (*14*,*15*).

## Conclusions

Although hantavirus infections in Canada remain rare, the high CFR and severe nature of the disease underscore the importance of surveillance-based awareness and risk prevention in at-risk populations and settings. Overall, close monitoring of SNV infections in Canada remains a key part of risk mitigation and will further our understanding of HCPS pathogenesis and SNV ecology and evolution.

Appendix Additional information on hantavirus cardiopulmonary syndrome in Canada.
