# Supplementary material for: Hantavirus Cardiopulmonary Syndrome in Canada
Source: Emerg Infect Dis. 2020 Dec;26(12):3020–4. doi: 10.3201/eid2612.202808 (PMC7706972; doi:10.3201/eid2612.202808)
Supplement: Appendix — Additional information on hantavirus cardiopulmonary syndrome in Canada. [file 20-2808-Techapp-s1.pdf]

Article DOI: https://doi.org/10.3201/eid2612.202808

Hantavirus Cardiopulmonary Syndrome in Canada

Appendix

Methods

Serology

Detection of IgG antibodies was done using an ELISA assay using Black Creek Canal orthohantavirus (BCCV)–infected VERO E6 lysate as a positive antigen and mock infected Vero E6 lysate as the negative antigen. The IgM detection assay is a mu-capture ELISA using BCCV-infected and mock-infected VERO E6 cell slurries and a mouse monoclonal antibody directed against aa 66-78 of the nucleoprotein of Sin Nombre orthohantavirus (SNV; United States Biologicals, https://www.usbio.net).

Molecular Detection

Patient samples positive for SNV were tested in a RT-qPCR using the primers: SNS140F 5′-AAKTGGACCCCGATGAYGTTAA and SNS140R 5′-TTGGTYTCCAATGCAGACACA and the probe SNS140P 6FAM-AAAAGCACATTACAGAGCAGACGGGCAG; we used the TaqPath 1-Step Multiplex Master Mix (Life Technologies, https://www.thermofisher.com) according to the manufacturer’s instructions.

To obtain sequence data for the small (S) and medium (M) genomic segments, we used 2 sets of primers. The original assay used was as described by Ksaizek et al. (*1*). Later the primers were optimized for SNV and the following sets were used. SNS2016F 5′-AGAAAGAGCRGTGGATGAYGTTAACA, SNS2016R 5′-GGACAACGATCSGATGCRAANACCCA, and SNM2016F 5′-CAAAAACAATGGTGTGTGAYATTTG, SNM2016R 5′-TTTATRTTGAATGCATCCATCCAATG. Amplicons were sequenced and used for phylogenic analyses. Sequences of sufficient length and quality were aligned along with full-length

reference sequences obtained from GenBank. Some sequences which aligned to a different region than most samples were removed from the alignment and analysis.

Phylogenetic Analysis

We ran 2 separate analyses, one for the S segment and one for the M segment. The sequences were aligned using MAFFT (https://mafft.cbrc.jp/alignment/software) with the --localpair option, a gap open penalty of 3, and a gap extension penalty of 1. The alignment was converted from FASTA to NEXUS format using seqmagick (https://fhcrc.github.io/seqmagick) (Appendix Table 1). We defined the BEAST model using the corresponding version of BEAUTi. The model used was Coalescent Constant model, the nucleotide model was the General Time-Reversible model (GTR), using a relaxed log-normal clock and empirical nucleotide frequencies. Each model (S & L) was run in 3 parallel chains for 20 million iterations each (logging every 1,000 iterations) using BEAST 2.5.2 (*2*). Convergence and effective sample sizes were confirmed using Tracer. The trees from the last 10 million iterations of each chain were combined using LogCombiner. Downsampled tree files were produced using LogCombiner to provide the uncertainty visualization (100 trees). The main posterior tree was derived using TreeAnnotator set to maximum clade credibility and common ancestor heights (with posterior probability limit of 0.95 and burn-in of 0 [burn-in was removed with Log Combiner]). Graphs were produced using R 3.6.1, with the following packages: checkpoint version 0.4.7 (snapshot date of 2019-11-11) (*3*), dplyr version 0.8.3 (*4*), tidyr version 1.0.0 (*5*), ggplot2 version 3.2.1 (*6*), ape version 5.3 (*7*), ggtree version 2.0.0 (*8*), cowplot version 1.0.0 (*9*), mapproj version 1.2.6 (*10*), ggmap version 3.0.0 (*11*), rnaturalearth version 0.1.0 (*12*), rnaturalearthhires version 0.2.0 (*13*), rgeos version 0.5-2 (*14*), and sf version 0.8-0 (*15*).

References

1. Ksiazek TG, Peters CJ, Rollin PE, Zaki S, Nichol S, Spiropoulou C, et al. Identification of a new North American hantavirus that causes acute pulmonary insufficiency. Am J Trop Med Hyg. 1995;52:117–23. PubMed https://doi.org/10.4269/ajtmh.1995.52.117

2. Bouckaert R, Vaughan TG, Barido-Sottani J, Duchêne S, Fourment M, Gavryushkina A, et al. BEAST 2.5: An advanced software platform for Bayesian evolutionary analysis. PLOS Comput Biol. 2019;15:e1006650. PubMed https://doi.org/10.1371/journal.pcbi.1006650

3. Ooi H. (2019). checkpoint: install packages from snapshots on the Checkpoint server for reproducibility. R package version 0.4.7 [cited 2020 Oct 14]. https://CRAN.R-project.org/package=checkpoint

4. Wickham H, François R, Henry L, Müller K. (2019). dplyr: a grammar of data manipulation. R package version 0.8.3 [cited 2020 Oct 14]. https://CRAN.R-project.org/package=dplyr

5. Hadley Wickham and Lionel Henry. (2019). tidyr: Tidy Messy Data. R package version 1.0.0 [cited 2020 Oct 14]. https://CRAN.R-project.org/package=tidyr

6. H. Wickham. ggplot2: elegant graphics for data analysis. New York: Springer-Verlag, 2016.

7. Paradis E, Schliep K. ape 5.0: an environment for modern phylogenetics and evolutionary analyses in R. Bioinformatics. 2019;35:526–8. PubMed https://doi.org/10.1093/bioinformatics/bty633

8. Guangchuang Y. Smith DK, Zhu H, Guan Y, Lam TT-Y. ggtree: an R package for visualization and annotation of phylogenetic trees with their covariates and other associated data. Methods Ecol Evol. 2017;8:28–36. https://doi.org/10.1111/2041-210X.12628

9. Claus O. Wilke (2019). cowplot: streamlined plot theme and plot annotations for ‘ggplot2’. R package version 1.0.0 [cited 2020 Oct 14]. https://CRAN.R-project.org/package=cowplot

10. McIlroy D. mapproj: Map Projections. R package version 1.2.6. 2018 [cited 2020 Oct 14]. https://CRAN.R-project.org/package=mapproj

11. Kahle D, Wickham H. ggmap: spatial visualization with ggplot2. The R Journal, 5, 144–161 [cited 2020 Oct 14]. http://journal.r-project.org/archive/2013-1/kahle-wickham.pdf

12. South A. rnaturalearth: world map data from Natural Earth. R package version 0.1.0. 2017 [cited 2020 Oct 14]. https://CRAN.R-project.org/package=rnaturalearth

13. South A. rnaturalearthhires: High resolution world vector map data from Natural Earth used in rnaturalearth. 2020 [cited 2020 Oct 14]. https://docs.ropensci.org/rnaturalearthhires

14. Bivand R, Rundel C. rgeos: interface to Geometry Engine - Open Source ('GEOS'). R package version 0.5–2. 2019 [cited 2020 Oct 14]. https://CRAN.R-project.org/package=rgeos

15. Pebesma E. Simple features for R: standardized support for spatial vector data. R J. 2018;10:439–46. https://doi.org/10.32614/RJ-2018-009

Appendix Table. GenBank accession numbers of reference sequences used in study of hantavirus cardiopulmonary syndrome, Canada

Virus name in trees	S segment accession no.	M segment accession no.
ConvictCreek_107	L33683.1	L33474.1
ConvictCreek_74	(Sequenced at NML)	L33684.1
NewYork	(Sequenced at NML)	U36801.1
77734	AF281851.1	AF281852.1
Isolate 2	JQ690282.1	——
BCC (Black Creek Canal)	L39949.1	L39950.1
BlueRiver	——-	AF030552.1

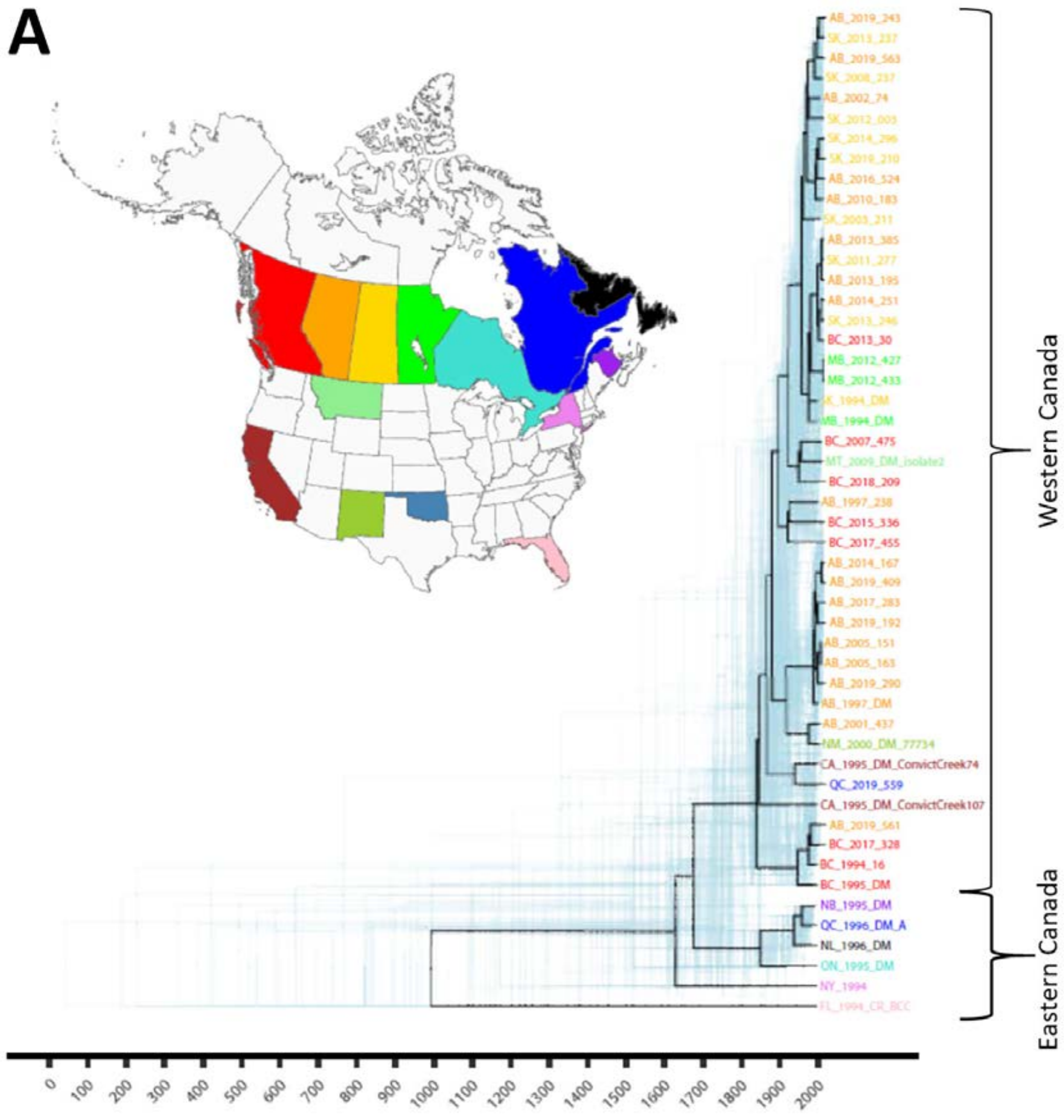

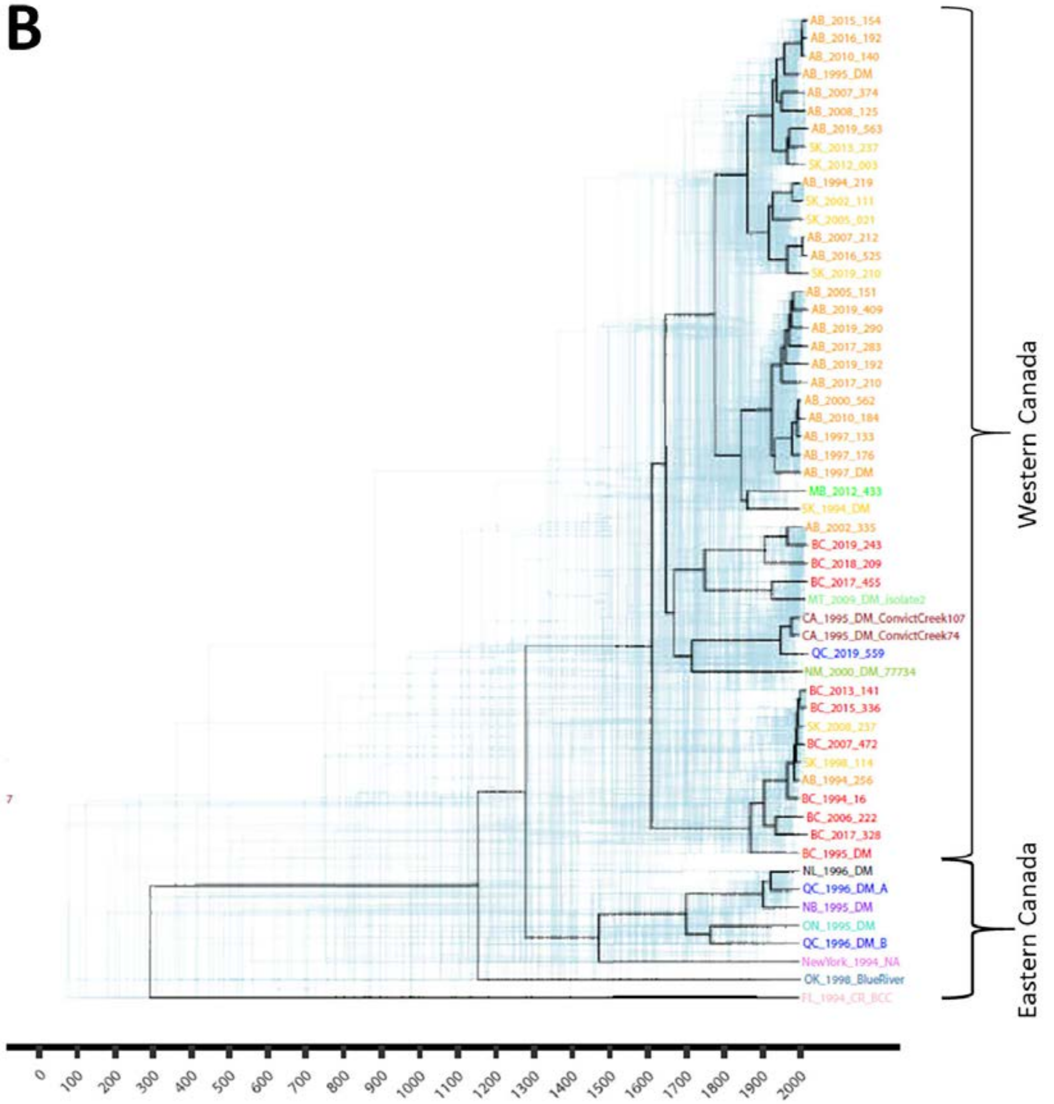
B
AB_2015_154
AB_2016_192
AB_2010_140
AB_1995_DM
AB_2007_374
AB_2008_125
AB_2019_563
SK_2013_237
SK_2012_003
AB_1994_219
SK_2002_111
SK_2005_021
AB_2007_212
AB_2016_525
SK_2019_210
AB_2005_151
AB_2019_409
AB_2019_290
AB_2017_283
AB_2019_192
AB_2017_210
AB_2000_562
AB_2010_184
AB_1997_133
AB_1997_176
AB_1997_DM
MB_2012_433
SK_1994_DM
AB_2002_335
BC_2019_243
BC_2018_209
BC_2017_455
MT_2009_DM_isolate2
CA_1995_DM_ConvictCreek107
CA_1995_DM_ConvictCreek74
QC_2019_559
NM_2000_DM_77734
BC_2013_141
BC_2015_336
SK_2008_237
BC_2007_472
SK_1998_114
AB_1994_256
BC_1994_16
BC_2006_222
BC_2017_328
BC_1995_DM
NL_1996_DM
QC_1996_DM_A
NB_1995_DM
ON_1995_DM
QC_1996_DM_B
NewYork_1994_NA
OK_1998_BlueRiver
FL_1994_CR_BCC
Western Canada
Eastern Canada
7
0
100
200
300
400
500
600
700
800
900
1000
1100
1200
1300
1400
1500
1600
1700
1800
1900
2000

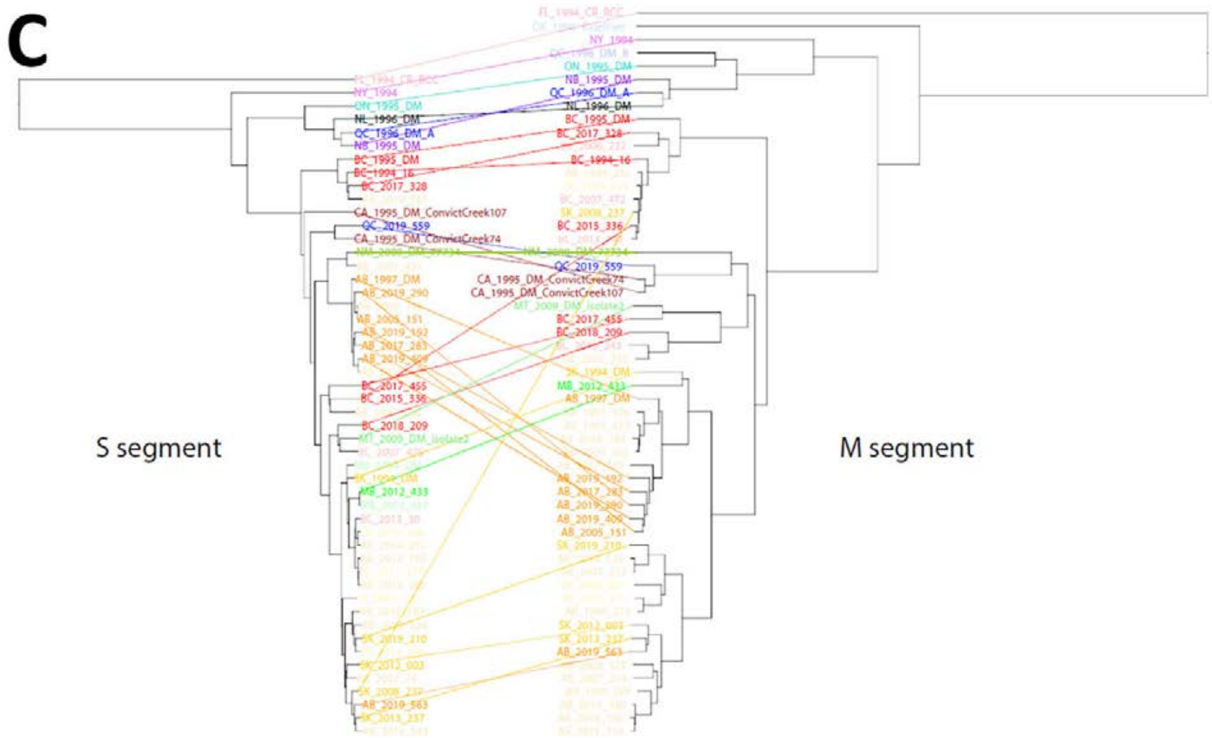

Appendix Figure. Molecular epidemiology and phylogenetic analysis of Sin Nombre virus (SNV) sequences from HCPS patients in Canada. A) Phylogenetic tree based on sequences of SNV small (S) segments from Canada and the United States. B) Phylogenetic tree based on sequences of SNV medium (M) segments from Canada and the United States. C) Corresponding S and M segments from Canadian HCPS patients. Lines connect S and M segments sequenced from the same SNV isolate.
